# Is progressive pneumoperitoneum useful in delayed repair of large omphaloceles?

**DOI:** 10.4103/0971-9261.43809

**Published:** 2008

**Authors:** Vivek Gharpure

**Affiliations:** Department of Pediatric Surgery, Children’s Surgical Hospital, Aurangabad, India

**Keywords:** Exomphalos major, pneumoperitoneum

## Abstract

Repair of exomphalos major in the neonatal period is fraught with risks and complications. Progressive pneumoperitoneum was found to be safe and cost-effective in six patients with exomphalos major who underwent repair at an older age. The technique is particularly suitable for hospitals that do not have facilities for intensive care, ventilation and total parenteral nutrition.

## INTRODUCTION

Exomphalos major affects 1:5000 live births. Loss of domicile by viscera causes difficulties in repair in the neonatal period. These babies often require ventilatory support, total parenteral nutrition (TPN) and intensive care after surgical repair.[1] These are not available in all hospitals in the country. We treated six patients with conservatively treated exomphalos major with preoperative progressive pneumoperitoneum, which avoided intensive care, ventilatory support and TPN, and yet led to a successful outcome.

## MATERIALS AND METHODS

Six patients with conservatively treated exomphalos major presented for repair of large ventral hernia. The age ranged from 18 to 90 months. There were four boys and two girls. All patients had a large skin-covered ventral hernia. Clinical examination did not reveal any other congenital anomaly. Routine investigations were within normal limits. A computed tomography (CT) scan of the abdomen showed presence of liver and small bowel in the hernial sac in all patients and a small abdominal cavity. The CT scan did not reveal abnormal hepatic veins. After explaining the procedure, written informed consent was obtained for the initial and definitive surgical procedures.

Patients underwent insertion of the peritoneal catheter under general anesthesia on day 1. A large bore polythene catheter was inserted in the peritoneal cavity in the left iliac fossa by the open method. The catheter was connected to a three-way canula and left closed.

The patient was allowed to recover from the anesthetic. Forty-eight hours after the insertion of the catheter, pneumoperitoneum was created by injection of air with a syringe and a three-way canula. Air was injected till the patient could tolerate the pressure. The process required 15–20 min. During and after pneumoperitoneum, heart rate, oxygen saturation and abdominal girth were measured. The patient was allowed oral fluids after 2 h and full feeds after 4 h. Pneumoperitoneum was created without general anesthetic in the ward. After 24 h, additional air was injected through the same catheter till the patient could tolerate the intraabdominal pressure. The process was continued for 7–9 days. Every day before injection of air, attempting to push the contents back in the peritoneal cavity and trying to bring the edges of defect together, reducibility of the hernial contents were assessed.

When enlargement of abdominal cavity was considered adequate, either clinically or radiologically the patient underwent a formal laparotomy. The pneumoperitoneal catheter was removed. All bowel and liver were separated from the sac and reduced into the peritoneal cavity. The abdominal cavity was found to be stretched adequately to accommodate the viscera. Straight-line closure without mesh could be achieved in all patients without undue tension. Routine postoperative care was administered. Oral feeds commenced after bowel function returned and patients were discharged after suture removal.

## RESULTS

All patients tolerated pneumoperitoneum very well; no patient had respiratory embarrassment requiring deflation of the abdomen. No patient was given total parenteral nutrition during the pneumoperitoneum phase or after repair. No patient required ventilatory support after repair of the large hernia. All patients could undergo anatomical repair of the abdominal wall. No patient required insertion of a prosthetic mesh in the abdominal wall. All patients survived. There were no infective or other complications.

## DISCUSSION

Many methods have been described for the repair of exomphalos major. Primary closure, ventilation and TPN are the most popular methods of repair. Several other techniques have been described for surgical repair, such as vacuum-assisted closure,[2] intraperitoneal tissue expander,[3] traction compression,[4] staged repair[5] and active enlargement in the neonatal period.[6] Some methods for delayed repair include pneumatic compression.[7] All these methods have complications in the neonatal period, complications of ventilation and complications of TPN. Skin closure creates adhesions between the bowel and the sac and makes delayed repair difficult. Mesh repair leaves a large foreign body in the tissues. Most require the baby to be kept in intensive care for a prolonged duration, investigations and monitoring, which may not be available in all hospitals in the country.

To obviate the complications and logistic difficulties of primary or delayed repair of exomphalos major, we used progressive pneumoperitoneum in these patients and found the technique eminently suitable for Indian conditions when ventilatory support, total parenteral nutrition and intensive care are not available in all hospitals.

Pneumoperitoneum has been described for large inguinal hernias in adults. The technique has been described for repair of giant inguinal hernias in adults.[8,9]

The advantages of this technique are that there is no need for ventilatory support, patients do not require total parenteral nutrition, there is no foreign body inserted in the tissues, blood loss is minimal, routine antibiotics are required, can be carried out in any public hospital without undue risk to the patient and is found to be safe.

The disadvantages are that upto 3 weeks are required for complete repair. If the patient has associated anomalies such as intestinal atresia, conservative management in the neonatal period may not be possible, the sac may rupture in the neonatal period and may require immediate repair. Vascular anomalies may preclude creation of pneumoperitoneum, which carries the risk of air embolism that can be eliminated with CO_2_ insufflation.
